# Structure of *Leishmania major* cysteine synthase

**DOI:** 10.1107/S1744309112019124

**Published:** 2012-06-22

**Authors:** Paul K. Fyfe, Gareth D. Westrop, Tania Ramos, Sylke Müller, Graham H. Coombs, William N. Hunter

**Affiliations:** a Division of Biological Chemistry and Drug Discovery, College of Life Sciences, University of Dundee, Dundee DD1 5EH, Scotland; b Strathclyde Institute of Pharmacy and Biomedical Sciences, University of Strathclyde, Glasgow G4 0RE, Scotland; c Institute of Infection, Immunity and Inflammation, Wellcome Trust Centre for Molecular Parasitology, College of Medical, Veterinary and Life Sciences, University of Glasgow, Glasgow G12 8TA, Scotland

**Keywords:** *Arabidopsis thaliana*, cysteine synthase, *Leishmania major*

## Abstract

Cysteine biosynthesis is a potential target for drug development against parasitic *Leishmania* species; these protozoa are responsible for a range of serious diseases. To improve understanding of this aspect of *Leishmania* biology, a crystallographic and biochemical study of *L. major* cysteine synthase has been undertaken, seeking to understand its structure, enzyme activity and modes of inhibition. Active enzyme was purified, assayed and crystallized in an orthorhombic form with a dimer in the asymmetric unit. Diffraction data extending to 1.8 Å resolution were measured and the structure was solved by molecular replacement. A fragment of γ-poly-d-glutamic acid, a constituent of the crystallization mixture, was bound in the enzyme active site. Although a d-­glutamate tetrapeptide had insignificant inhibitory activity, the enzyme was competitively inhibited (*K*_i_ = 4 µ*M*) by DYVI, a peptide based on the C-­terminus of the partner serine acetyltransferase with which the enzyme forms a complex. The structure surprisingly revealed that the cofactor pyridoxal phosphate had been lost during crystallization.

## Abstract 2

A crystallographic and biochemical study of *L. major* cysteine synthase, which is a pyridoxyl phosphate-dependent enzyme, is reported. The structure was determined to 1.8 Å resolution and revealed that the cofactor has been lost and that a fragment of γ-poly-d-glutamic acid, a crystallization ingredient, was bound in the active site. The enzyme was inhibited by peptides.

## 1. Introduction

*Leishmania*, a widespread and important protozoan pathogen of humans and animals, requires cysteine for protein biosynthesis and as a precursor of trypanothione, a glutathione–spermidine conjugate unique to trypanosomatids with an essential role in redox metabolism and antioxidant defence (Krauth-Siegel & Comini, 2008 ▶). Cysteine is also the source of reduced sulfur for the biosynthesis of important metabolites such as coenzyme A, enzyme cofactors and iron–sulfur clusters (Nozaki *et al.*, 2005 ▶). The vital role of cysteine raises the questions of how *Leishmania* obtains the amino acid, how cysteine metabolism in *Leishmania* might differ from that in the mammalian host and whether such differences might be targeted in drug-discovery research. *L. major* does not have a high-affinity transporter for the uptake of cysteine, but it can acquire methionine and, like the mammalian host, it has the enzymes required to convert methionine to cysteine by transsulfuration (Williams *et al.*, 2009 ▶). The parasite can also produce cysteine from serine in a two-step process (Williams *et al.*, 2009 ▶). Firstly, serine acetyltransferase (SAT) generates *O*-acetylserine (OAS) to supply the substrate for the second stage, which is catalyzed by the pyridoxal phosphate (PLP)-dependent cysteine synthase (CS; EC 2.5.1.47). This *de novo* pathway for cysteine biosynthesis is found in plants, bacteria and some protozoa, but is absent from mammals. In principle, *L. major* CS (*Lm*CS) may represent a drug target, and an improved understanding of the enzyme might usefully inform on its potential in this respect. In particular, knowledge of the structure can support the development of reagents to chemically validate the target or to provide early-stage information on inhibitors (Hunter, 2009 ▶).

Some types of CS, including bacterial *O*-acetylserine sulfhydrylase type A (OASS-A) and plant *O*-acetylserine thiol-lyase (OAS-TL), combine reversibly with SAT to form a bi-enzyme complex in which SAT is active and CS is strongly inhibited (Campanini *et al.*, 2005 ▶). The substrates of CS are effectors of complex formation; the complex is dissociated by elevated levels of OAS but is stabilized by sulfide. The complexes formed in plants and bacteria have distinctive features that indicate different regulatory functions (Salsi, Campanini *et al.*, 2010 ▶; Wirtz *et al.*, 2010 ▶). It has been established that the C-terminal end of SAT is critical for its interaction with CS and, in particular, all SATs possess a C-terminal isoleucine which is essential for CS binding. Peptides corresponding to the C-terminus of SAT bind to the active site of CS and structural data have revealed that the carboxyl­ate group of the C-terminal isoleucine occupies the same space and makes the same interactions as the carboxylate of the α-aminoacrylate catalytic intermediate formed after β-elimination of acetate from the substrate OAS (Rabeh & Cook, 2004 ▶; Huang *et al.*, 2005 ▶; Francois *et al.*, 2006 ▶; Schnell *et al.*, 2007 ▶; Salsi, Bayden *et al.*, 2010 ▶). A four-amino-acid SAT peptide has been shown to be a competitive inhibitor of *Mycobacterium tuberculosis* CS with a *K*_i_ of 5 µ*M*, providing a simple mechanism for complex formation and its dissociation in the presence of elevated levels of OAS (Schnell *et al.*, 2007 ▶). Sequence alignments indicate that *Lm*CS contains a SAT-binding motif that was originally identified in *Arabidopsis thaliana* OAS-TL (*At*OAS-TL; Bonner *et. al.*, 2005 ▶) and the enzyme can also bind SAT when the proteins are co-expressed in *Escherichia coli* (Williams *et al.*, 2009 ▶).

We undertook a crystallographic and biochemical study of *Lm*CS to investigate the interactions of the enzyme with ligands, including potential inhibitors. Our overall aim was to improve understanding of the enzyme in *Leishmania* and to provide information that might help to assess the potential of CS as a target for structure-based approaches to develop inhibitors with suitable chemical properties to underpin early-stage drug discovery (Hunter, 2009 ▶).

## 2. Materials and methods

### 2.1. Protein expression, purification and crystallization

The recombinant *E. coli* expression system for *Lm*CS (Williams *et al.*, 2009 ▶) was modified by subcloning the *LmCS* gene from vector pET21a+ into pET15bTEV to allow production of an N-terminally His-tagged protein, which was purified following a standard protocol (Bond *et al.*, 2001 ▶). Briefly, the first stage involved nickel ion-affinity chromatography through a 5 ml Ni–NTA column (Qiagen). The product was eluted in a linear imidazole-concentration gradient, which was followed by incubation for 2 h with His-tagged tobacco etch virus (TEV) protease at 303 K prior to dialysis at room temperature against 20 m*M* Tris–HCl, 150 m*M* NaCl pH 7.5 for 1 h. The resulting mixture was reapplied onto the Ni–NTA column, which binds the cleaved His tag, the TEV protease and any remaining uncleaved *Lm*CS. The *Lm*CS from which the His tag had been cleaved was present in the flowthrough. Fractions were analyzed using SDS–PAGE and those containing *Lm*CS were pooled. The protein was further purified by size-exclusion chromatography using a Superdex 200 26/60 column (GE Healthcare) equilibrated with 20 m*M* Tris–HCl, 150 m*M* NaCl pH 7.5. The final level of *Lm*CS purity was confirmed by matrix-assisted laser desorption/ionization-time of flight mass spectrometry. In preparation for crystallization, the sample was dialyzed into 10 m*M* Tris–HCl, 100 m*M* NaCl pH 7.8 and concentrated using a Vivaspin 20 (Sartorius) to provide a stock solution for crystallization. A theoretical extinction coefficient of 16 180 *M* ^−1^ cm^−1^ at 280 nm was used to estimate protein concentration (*ProtParam*; Gasteiger *et al.*, 2005 ▶); the theoretical mass of one subunit is estimated as 35.6 kDa.

Crystallization was achieved at 293 K using the hanging-drop vapour-diffusion method with 0.75 µl protein solution at a concentration of 10 mg ml^−1^ mixed with 0.75 µl reservoir solution consisting of 7.5% PGA-LM (γ-poly-d-glutamic acid low molecular weight) and 19% PEG 3350 (polyethylene glycol average mass 3350) in 0.1 *M* Tris–HCl pH 7.8. Crystals grew over a period of 2–3 d to approximate dimensions of 50 × 50 × 250 µm and were characterized in-house using a Rigaku HF007 rotating-anode X-ray generator coupled to an R-AXIS IV^++^ image-plate detector. The presence of PGA-LM and PEG 3350 in the mother liquor allowed the crystals to be cooled to approximately 103 K in a stream of gaseous nitrogen without additional cryoprotection. The crystals were orthorhombic and belonged to space group *P*2_1_2_1_2_1_, with unit-cell parameters *a* = 48.96, *b* = 86.3, *c* = 134.0 Å. Suitable crystals were stored in liquid nitrogen for subsequent data collection at the European Synchrotron Radiation Facility (ESRF), Grenoble, France.

### 2.2. X-ray data collection, processing, structure solution and refinement

A well formed sample was selected and diffraction data were measured on beamline ID23-2 at the ESRF using a MAR 225 CCD detector. Data were indexed and integrated using *XDS* (Kabsch, 2010 ▶) and scaled using *SCALA* (Evans, 2006 ▶); the statistics are summarized in Table 1 ▶. Diffraction data were collected from a single crystal at a wavelength of 0.87260 Å. The search model for molecular replacement was prepared from the *E. coli* cysteine synthase B structure (PDB entry 2bhs; Claus *et al.*, 2005 ▶). The sequence identity between the search model and *Lm*CS is 39%. Pruning and mutation of this model was carried out using *CHAINSAW* (Stein, 2008 ▶). Molecular replacement was performed in *MOLREP* (Vagin & Teplyakov, 2010 ▶) using a monomer from 2bhs to search for two molecules in the asymmetric unit. A dimer was located, giving a score of 0.396. Refinement was performed in *REFMAC*5 (Murshudov *et al.*, 2011 ▶) and was alternated with rounds of electron-density and difference density map inspection and model manipulation together with water and ligand incorporation using *Coot* (Emsley & Cowtan, 2004 ▶). *MolProbity* (Chen *et al.*, 2010 ▶) was used to investigate model geometry in combination with the validation tools provided in *Coot*. Final model analysis was performed using *JCSG Quality Control Check* (http://smb.slac.stanford.edu/jcsg/QC/). Crystallographic statistics are presented in Table 1 ▶. Analyses of surface areas and interactions were made using the *PISA* server (Krissinel & Henrick, 2007 ▶) and the figures were prepared with *PyMOL* (DeLano, 2002 ▶). Amino-acid sequence alignments were carried out using the program *MUSCLE* (Edgar, 2004 ▶).

### 2.3. Biochemical analysis

For biochemical analysis, recombinant *Lm*CS was expressed and purified as a C-terminally His-tagged protein as described previously (Williams *et al.*, 2009 ▶). The *A. thaliana OAS-TL* gene was subcloned from pET3dAtOASTL into pET21 and the recombinant protein *At*OAS-TL was expressed and purified in the same way as *Lm*CS.

CS activities were determined at room temperature in 100 µl 200 m*M* potassium phosphate, 1 m*M* EDTA, 0.2 m*M* PLP, 1 mg ml^−1^ BSA, 3 m*M* OAS, 2 m*M* sodium sulfide pH 7.8 with 8 ng *Lm*CS or 12 ng *At*OAS-TL. The reaction was started by the addition of sodium sulfide after incubation of all other components for 5 min. Samples were taken before addition of sodium sulfide (0 min) and then every 2 min for 10 min; the cysteine produced was quantified using the azo-dye method described previously (Williams *et al.*, 2009 ▶). The rates of cysteine production were linear for 10 min and the specific activities obtained for *Lm*CS and *At*OAS-TL were 180 ± 18 and 130 ± 20 µmol min^−1^ mg^−1^, respectively. C-terminal SAT peptides are known to bind to the active sites of the plant OAS-TL (Francois *et al.*, 2006 ▶) and bacterial OASS enzymes (Huang *et al.*, 2005 ▶) and a peptide DFSI based on the SAT sequence is a competitive inhibitor of *M. tuberculosis* OASS (Schnell *et al.*, 2007 ▶). Thus, peptides based on the *A. thaliana* and *L. major* SATs and the PGA bound in the crystal of *Lm*CS were tested as inhibitors of the enzyme. Inhibition data were determined by adding various concentrations of different peptides to the pre-incubation mixture and then measuring the enzyme activity. IC_50_ curves were obtained using *GraFit* 5 (Erathicus) by plotting the initial rates measured with at least six different concentrations of the peptide. All IC_50_ values are the means ± standard deviations of three independent determinations, unless otherwise stated. The kinetics of inhibition by the tetrapeptide DYVI were investigated by measuring the initial rates of *Lm*CS without the peptide and then with four different concentrations of peptide (10–100 µ*M*) and six different concentrations of OAS (2.5–20 m*M*) at a fixed concentration of 2.0 m*M* sodium sulfide. The type of inhibition was determined from the pattern of the double-reciprocal plots of 1/*V* against 1/[*S*] for the different peptide concentrations. The *K*_i_ was determined by replotting the slopes against the peptide concentration which for competitive inhibition is linear, with the intersect on the *x* axis representing −*K*_i_.

## 3. Results and discussion

### 3.1. General comments and overall *Lm*CS structure

**Figure 1:**
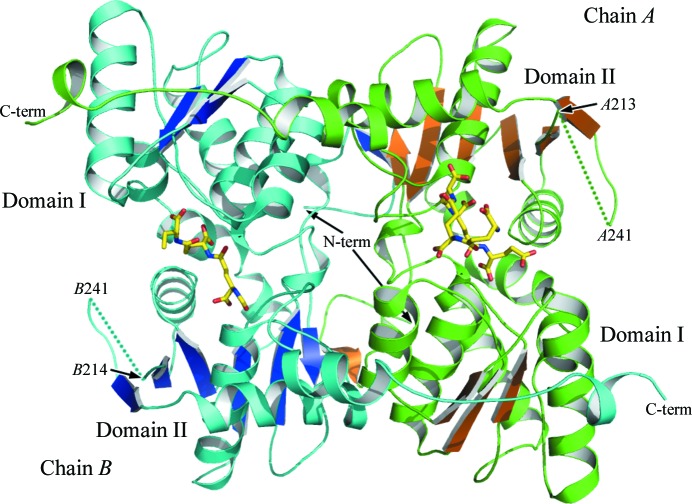
A ribbon diagram of the *Lm*CS dimer. The helices and strands of subunit *A* are coloured green and brown, respectively, and in subunit *B* they are coloured cyan and blue, respectively. The position of the disordered loops *A*214–*A*241 and *B*213–*B*241 are marked by dotted lines. The C- and N-terminal positions are labelled, as are the domains. The two molecules of γ-poly-d-glutamic acid are depicted in stick form, with C positions coloured yellow, O positions red and N positions blue.

The structure of *Lm*CS was determined to a resolution of 1.8 Å. The biologically active unit, a dimer, constitutes the asymmetric unit (Fig. 1 ▶). Subunit *A* contains residues 3–213 and 241–333, whilst subunit *B* comprises residues 4–214 and 241–333. A surface loop from residues 214 to 241 is disordered and is therefore missing from the model. The *Lm*CS subunit contains two domains. The smaller domain I is constructed by residues 51–158, which primarily form a four-stranded β-sheet surrounded by four α-helices. The larger domain II comprises residues 21–50 and 159–306. Domain II contains four α-­helices and six β-strands which, together with a β-strand contributed from the partner-subunit domain I, form a seven-membered β-­sheet. In addition, residues 307–333 at the C-terminus form an extended helix–loop–helix structure that stretches across the surface of the partner subunit. This extension is positioned on the opposite face of the dimer to that of the β-sheet intersubunit interaction. These two areas make major contributions to the area of the dimerization interface, which constitutes 22% or 3280 Å^2^ of the surface area of each subunit.

The enzyme purified from the *E. coli* expression host was cata­lytically active and displayed a yellow colour. Both observations are consistent with the presence of the PLP cofactor. In addition, PLP was added prior to crystallization, seeking to ensure full occupancy. However, the crystals were colourless and there was no electron density to indicate that PLP was present. The affinity of the crystallization agent PGA-LM to bind to *Lm*CS may contribute to the loss of PLP that is observed and the position of a loop formed by residues 181–190 is likely to be a consequence of the absence of the cofactor.

### 3.2. Binding of γ-poly-d-glutamic acid

A fragment of the crystallization agent PGA-LM is bound in an ordered fashion to the same region of both subunits of the *Lm*CS dimer. PGA is a pseudopeptide comprising d-glutamic acid residues linked through the amide N atom and the γ-carboxy O atom of an adjacent unit. The use of this compound in protein crystallization was highlighted by Hu *et al.* (2008 ▶).

**Figure 2:**
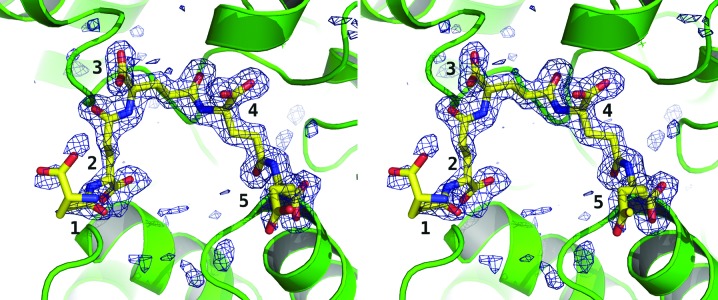
Stereoview of the PGA fragment bound to subunit *A*. An *F*_o_ − *F*_c_ OMIT difference density map is shown, where *F*_o_ are the observed and *F*_c_ are the calculated structure factors derived from the crystallographic model excluding the contributions from PGA atoms. The map is contoured at 3σ (blue chicken wire) and PGA is shown as a stick model with C positions coloured yellow, O positions red and N positions blue. The protein is depicted in a green ribbon format. The d-glutamic acid units are numbered 1–5.

**Figure 3:**
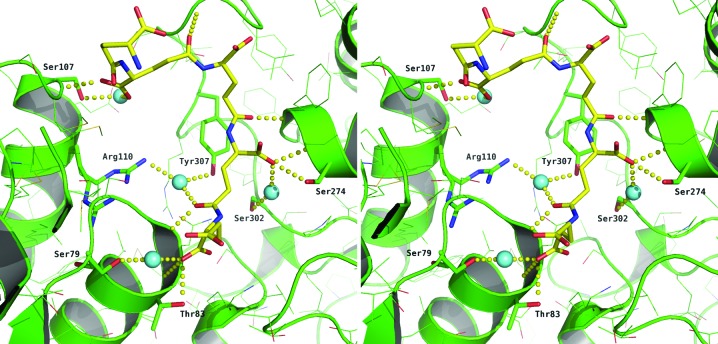
Stereoview of the binding of PGA-*A* to *Lm*CS. PGA is shown as in Fig. 1 ▶ and *Lm*CS is shown in green, with N and O atoms of specific side chains coloured blue and red, respectively. Water molecules are shown as cyan spheres and hydrogen-bonding interactions are depicted as yellow dotted lines.

In subunit *A* PGA-*A* comprises five d-glutamic acid moieties (Fig. 2 ▶), while PGA-*B* consists of three d-glutamic acids bound to subunit *B* (data not shown). The first and second glutamate moieties of PGA-*A* overlap with the second and third glutamates of PGA-*B* (data not shown). PGA-*A* is extended by three additional moieties at one end, while PGA-*B* has an additional moiety at the other end of the ligand. The interactions between carboxyls from PGA and the side chain and main chain of Thr83 and the main-chain amides of Asn82, Ser274 and Phe273 are common to both binding sites. Also involved in binding PGA-*A* are Leu312, Ala311 and Ser107 (Fig. 3 ▶), while binding of PGA-*B* also involves Ser78, Ser80, Arg110 and Thr101 (data not shown).

### 3.3. Comparisons with *At*OAS-TL and the PLP-binding site

**Figure 4:**
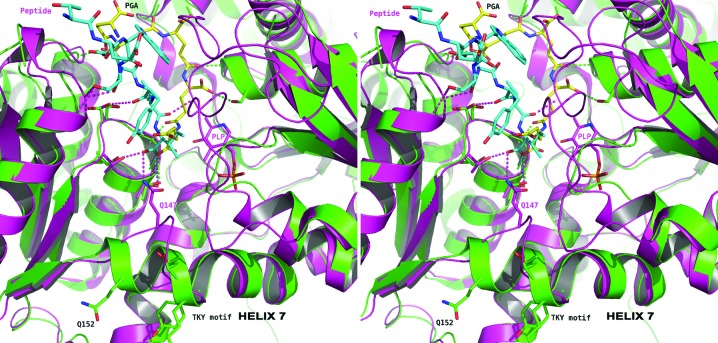
Stereoview of the PGA-*A*–*Lm*CS complex overlaid with a peptide–*At*OAS-TL complex. The *Lm*CS structure is shown as in Fig. 3 ▶, with the locations of α7 and the TKY motif indicated. The model of the *At*OAS-TL–peptide complex (PDB entry 2isq; Francois *et al.*, 2006 ▶) is coloured purple. The peptide sequence corresponds to that of the C-­terminal residues of SAT. Hydrogen-bonding interactions are depicted as dashed lines coloured according to the structure in which they occur.

*Lm*CS shows a high level of sequence identity to other *O*-acetyl­serine sulfhydrylases in the PDB. Analysis of the structural conservation using *DALI* (Holm & Rosenström, 2010 ▶) revealed the highest similarity to be to *At*OAS-TL (PDB entry 1z7w; Bonner *et al.*, 2005 ▶), which shares 47% sequence identity. The superimposition of subunits with *Lm*CS gives an r.m.s.d. of 0.9 Å over 285 C^α^ residues. Differences between the structures are primarily restricted to loops positioned around the active site. The region from Asp151 to Tyr157 in *Lm*CS forms the start of α7, while in *At*OAS-TL this helix is truncated (Fig. 4 ▶). The conserved motif QF*X*NP*X*N that is present in the vast majority of OAS-TL sequences is replaced by QFATKYN in *Lm*CS; this replacement is also found in *L. braziliensis*, *L. infantum* and *Trypanosoma cruzi*. The first residue of this motif, glutamine, is normally directed towards the active site, although it remains too distant to interact directly with the cofactor. Alteration to the TKY motif causes restructuring of this region, extending the helix that normally follows the motif by an extra two turns. This has two effects. Firstly, the glutamine (Gln152) residue is placed on the opposite face of the helix, far removed from the active site. In addition, the phenylalanine (Phe153) is also positioned away from the entrance to the active site. In *Lm*CS, the placement of these two residues ensures that the active site is considerably widened with respect to that found in orthologues of known structure.

The high level of structural conservation between *Lm*CS and the existing structures of *O*-acetylserine sulfhydrylases is such that the expected binding position for the cofactor PLP can be reliably derived. A lysine (Lys51 in *Lm*CS) forms a Schiff base with PLP, with a conserved asparagine and serine, Asn82 and Ser274 in *Lm*CS, forming hydrogen bonds to PLP. Further residues predicted to orient and hold the PLP in position are Gly186, Thr182, Gly183 and Thr185 of *Lm*CS, which are strictly conserved as Gly181, Thr187, Gly188 and T190 in *At*OAS-TL. Structural differences are observed between *Lm*CS and *At*OAS-TL in the loop formed by residues 184–190. This loop is glycine-rich; it is therefore likely to be mobile and its position in *Lm*CS is probably influenced by the absence of PLP from the active site.

The binding of PGA may have contributed to the absence of PLP and the unresolved loop between residues 214 and 241 in *Lm*CS (Fig. 4 ▶). Again comparing *Lm*CS with *At*OAS-TL, it is expected that this loop would fold down and bind into the same groove on the CS surface as that occluded by PGA (Fig. 4 ▶). Closer analysis of the position of this loop in *At*OAS-TL and comparison with the *Lm*CS structure reveals that PGA interacts with Ser274 (Fig. 3 ▶) and supplants the interactions normally expected to form when PLP is bound in the active site (Fig. 4 ▶). The position of the side chain of the conserved Arg110 changes considerably as it forms interactions with PGA, whereas normally it would be expected to interact with the highly conserved Gln224 when the 214–241 loop closes over the active site.

### 3.4. Biochemical analysis

Following the initial observation of PGA binding in the active site, we tested a (γ-Glu)_4_ derivative as a potential inhibitor. Despite testing up to a concentration of 500 µ*M*, we were unable to detect any significant inhibition (data not shown). This may relate to the finding, described above, that PGA binding to *Lm*CS does not mimic that of peptides to the active PLP-containing enzyme and may simply be a consequence of the high level of PGA present in the crystallization conditions.

**Figure 5:**
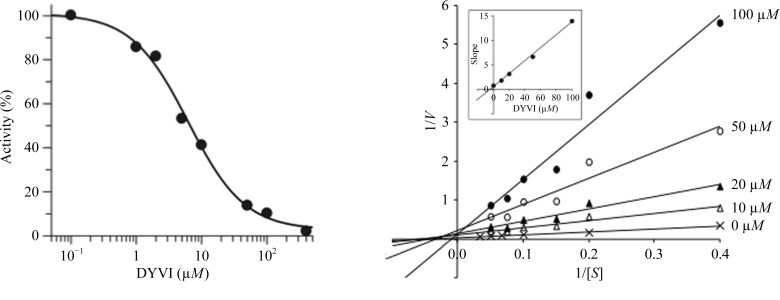
Inhibition of *Lm*CS by the synthetic peptide DYVI. (*a*) Dose-response curve. Initial rates were determined in reactions containing 3 m*M* OAS and eight different concentrations of the peptide DYVI (0.1–400 µ*M*). The graph shows percentage activity plotted against concentration of the peptide and the IC_50_ curve fitted with *GraFit* 5. (*b*) Double-reciprocal plots of initial velocity against substrate concentration showing competitive inhibition. Reactions contained four different concentrations of the peptide (10–100 µ*M*) with six different concentrations of the substrate OAS (2.5–20 m*M*). Lines were plotted for each peptide concentration by linear regression using *Microsoft Excel*. Reactions contained 100 µ*M* DYVI (closed circles), 50 µ*M* DYVI (open circles), 20 µ*M* DYVI (closed triangles), 10 µ*M* DYVI (open triangles) or no inhibitor (crosses). The inset is the secondary plot of slope values against the concentration of DYVI.

Peptides corresponding to the C-terminus of the *L. major* and *A. thaliana* SATs were also tested as inhibitors of *Lm*CS (Table 2 ▶). The results obtained for the plant SAT peptide (DYVI), which inhibited *Lm*CS with an IC_50_ of 7 µ*M* and displayed a similar activity towards *A. thaliana* OAS-TL, are presented in Fig. 5 ▶. Surprisingly, the *Leishmania* C-terminal peptide GSGI was only a weak inhibitor, with an IC_50_ of approximately 1.5 m*M*. Longer peptides derived from the *Leishmania* SAT sequence had greater activity, with the C-terminal heptapeptide (EGDGSGI) inhibiting *Lm*CS with an IC_50_ of 270 µ*M*. Similar results were obtained when the *Leishmania* SAT peptides were tested on *At*OAS-TL. These findings are consistent with data from the mutational analysis of SAT from *E. coli*, which showed that both length and the presence of negatively charged residues were important for complex formation (Zhao *et al.*, 2006 ▶). Attempts to cocrystallize *Lm*CS with DYVI were unsuccessful; thus, the binding could not be analysed further.

Heterologous binding of divergent SAT peptides has been reported previously (Campanini *et al.*, 2005 ▶; Francois *et al.*, 2006 ▶). The *K*_i_ of the *A. thaliana* SAT peptide DYVI for *Lm*CS was determined by measuring the activity with different concentrations of OAS and the peptide at a fixed concentration of sodium sulfide. The double-reciprocal plots showed that the apparent *K*_m_ increased with increasing peptide concentration, but the *V*_max_ was not altered and the secondary plot of slope against peptide concentration was linear (data not shown). These results indicate that the heterologous SAT peptide DYVI is a competitive inhibitor of *Lm*CS, with a *K*_i_ of 4 µ*M*, similar to the inhibition reported for the *M. tuberculosis* enzyme by its cognate SAT peptide (Schnell *et al.*, 2007 ▶).

The carboxylate group of the invariant C-terminal isoleucine provides an anchor for peptide binding and forms hydrogen bonds between key active-site residues that also bind the substrate OAS. These interactions are highly conserved between species (Francois *et al.*, 2006 ▶; Schnell *et al.*, 2007 ▶). These are in part conserved in the PGA binding through Thr83, but the carboxylate lies too distant from Ser79 to conserve the second interaction (Fig. 3 ▶).

Given the importance of the C-terminal isoleucine residue, the free amino acid and certain N-blocked derivatives were also tested for inhibition (Table 2 ▶). Isoleucine itself had no activity at 4 m*M*, whereas two of the N-blocked derivatives inhibited with IC_50_ values of 250 µ*M*. This increased activity could be the result of the removal of the positively charged amino group or the addition of the hydrophobic blocking group. The carboxybenzyl (CBZ) blocked dipeptide CBZ-l-­valine-isoleucine showed a greatly reduced activity compared with other blocked amino acids, indicating that a valine at this position of the peptide may not be optimal.

The relatively weak activity of the *Leishmania* SAT peptides for competitive inhibition of *Lm*CS raises questions as to whether CS and SAT could form a functional protein–protein complex in this organism. Differences in the dissociation constants observed for plant and bacterial cysteine synthase complexes (CSCs) were thought to result from differences in the affinity of the SAT C-terminus for the CS active site (Wirtz *et al.*, 2010 ▶). However, complex formation in plants and bacteria is now known to involve conformational changes in CS that are not induced by binding of the C-terminal SAT peptide alone (Campanini *et al.*, 2005 ▶; Salsi, Campanini *et al.*, 2010 ▶; Wirtz *et al.*, 2010 ▶; Kumaran *et al.*, 2009 ▶). Additional interactions between SAT and CS appear to be required. A sequence motif that was first identified in *At*OAS-TL has been implicated in complex formation with SAT by mutagenesis of several conserved basic residues (Lys217, His221 and Lys222) in *At*OAS-TL (Bonner *et al.*, 2005 ▶), and mutation of the corresponding residues (Lys222, His226 and Lys227) in *Lm*CS also prevented complex formation when CS and SAT were co-expressed in *E. coli* (Williams *et al.*, 2009 ▶). These residues are located on the disordered 213–241 loop in *Lm*CS and are therefore well placed to bind a partner protein at the active site. Although it has been predicted on the basis of molecular modelling that the C-­terminal sequence of *Leishmania* SAT would not be able to bind to the CS active site (Marciano *et al.*, 2010 ▶), our inhibition data, and the conservation of basic residues shown to contribute to complex formation, suggest that *L. major* SAT and CS could indeed interact. The relatively weak initial interaction with the C-terminal isoleucine might precede conformational changes that support complex formation as observed in the assembly of bacterial CSCs (Salsi, Bayden *et al.*, 2010 ▶; Wang & Leyh, 2012 ▶). Further work would be required to investigate this aspect of *Lm*CS function, although the presence of PGA in the active site and the loss of PLP cofactor indicate that these crystallization conditions and the crystal form obtained in this study are unsuited to a study of *Lm*CS complexes.

## Supplementary Material

PDB reference: cysteine synthase, 4air

## Figures and Tables

**Table 1:** Data-collection and refinement statistics Values in parentheses are for the highest resolution bin of approximate width 0.1 Å.

Data collection	
Space group	*P*2_1_2_1_2_1_
Unit-cell parameters (Å)	*a* = 48.9, *b* = 86.3, *c* = 134.0
Resolution range (Å)	45–1.8
Unique reflections	53553
Completeness (%)	100 (100)
〈*I*/σ(*I*)〉	18.0 (3.5)
Multiplicity	6.0 (6.0)
*R*_merge_ † (%)	7.1 (48.8)
Refinement	
No. of reflections used	50629
*R*_work_ ‡ (%)	15.7
*R*_free_ § (%)	20.8
Protein atoms	5140
Molecules and ions present	
Water	700
PGA	2
Cl^−^	2
R.m.s. deviations from ideal geometry	
Bond lengths (Å)	0.015
Bond angles (°)	1.44
Thermal parameters (Å^2^)	
Wilson *B*	18.4
Mean *B*	
Protein	19.3
Water	33.1
PGA	33.4
Cl^−^	15.7
Ramachandran plot¶ (%)	
Favoured	97.2
Allowed	2.8

† *R*_merge_ = 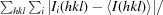 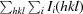.

‡ *R*_work_ = 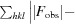 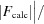 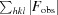, where *F*_obs_ is the observed structure factor and *F*_calc_ is the calculated structure factor.

§ *R*_free_ is the same as *R*_work_, except calculated using 5% of the data that were not included in any refinement calculations.

¶ Ramachandran analysis from *Coot*.

**Table 2:** Inhibition of *Lm*CS by peptides corresponding to the C-termini of the *L. major* and *A. thaliana* SATs Results in bold represent the mean ± standard deviation of three independent determinations. All other results are from a single IC_50_ curve. ND, not determined.

	*At*OAS-TL IC_50_ (µ*M*)	*Lm*CS IC_50_ (µ*M*)
Peptides		
DYVI	**8 ± 3**	**7 ± 1**
GSGI	≤2000	1500
DGSGI	170	**620 ± 50**
EGDGSGI	240	**270 ± 70**
N-blocked amino acids		
L-Isoleucine	ND	>4000
*N*-Benzyl-L-isoleucine	ND	>1000
*N*-3-Indolylacetyl-L-isoleucine	ND	260
*N*-CBZ-L-isoleucine	ND	320
*N*-CBZ-L-valine-isoleucine	ND	1410
